# Spatial analogies pervade complex relational reasoning: Evidence from spontaneous gestures

**DOI:** 10.1186/s41235-016-0024-5

**Published:** 2016-12-07

**Authors:** Kensy Cooperrider, Dedre Gentner, Susan Goldin-Meadow

**Affiliations:** 1 Department of Psychology, University of Chicago, 5848 S. University Avenue, Chicago, IL 60637 USA; 2 Department of Psychology, Northwestern University, 2029 Sheridan Road, Evanston, IL 60208 USA

**Keywords:** Analogy, Relational reasoning, Gesture, Complex systems, Spatial cognition

## Abstract

How do people think about complex phenomena like the behavior of ecosystems? Here we hypothesize that people reason about such relational systems in part by creating spatial analogies, and we explore this possibility by examining spontaneous gestures. In two studies, participants read a written lesson describing positive and negative feedback systems and then explained the differences between them. Though the lesson was highly abstract and people were not instructed to gesture, people produced spatial gestures in abundance during their explanations. These gestures used space to represent simple abstract relations (e.g., *increase*) and sometimes more complex relational structures (e.g., *negative feedback*). Moreover, over the course of their explanations, participants’ gestures often cohered into larger analogical models of relational structure. Importantly, the spatial ideas evident in the hands were largely unaccompanied by spatial words. Gesture thus suggests that spatial analogies are pervasive in complex relational reasoning, even when language does not.

## Significance

Many phenomena in the natural and social world involve complex causal systems. Biological organisms, financial markets, ecosystems, and mechanical devices, for instance, all exhibit different types of feedback relationships, in which multiple causal factors in a system change and bring about changes in each other. Feedback systems are central to the STEM fields, as well as to urgent societal issues such as climate change. Here we investigate the possibility that people reason about such causal patterns in part by using *spatial analogies*—that is, spatial models of the relations involved. To investigate this possibility, we had people explain the differences between positive and negative feedback systems and examined their gestures. Gesture is ubiquitous in communication and classroom instruction, and it sometimes expresses spatial ideas not found in speech. We found that people gestured copiously when explaining the feedback systems, even though such systems are not inherently spatial. For example, people located different elements of the system in different parts of space, showed causal relationships as movements between locations, and modeled the overall behavior of the systems with complex movements. Interestingly, most of these spatial gestures were produced without spatial words. These findings suggest that spatial analogies may be a pervasive part of people’s on-the-fly reasoning about complex relational systems, and, further, that gesture may be an important medium through which such analogies are expressed. The pervasiveness of spatial analogy in reasoning about complex phenomena has implications for how such phenomena are taught in a range of fields.

## Backround

Permafrost once covered much of the Arctic, but it is now thawing. This has a number of consequences, but one is especially insidious: as the frozen ground melts it releases methane, which then enters the atmosphere and accelerates the very warming that caused the ground to thaw in the first place (Anthony, 2009). This self-reinforcing pattern is an example of a *complex causal system*: multiple causal factors—in this case, atmospheric temperature, melting permafrost, and methane release—are changing over time and are causing changes in each other. Such systems are legion in the environmental sciences, but are hardly limited to them. Further examples can be found in financial markets, in the human body, in household appliances, in plant physiology, and beyond. Indeed, complex relational systems underlie phenomena throughout the natural and social world, in all domains and on all scales. Yet, despite the ubiquity of such systems—and their centrality to STEM (Science, Technology, Engineering, and Mathematics) fields and to urgent societal issues—much remains to be learned about the cognitive processes involved in understanding them. To fully grasp the alarming implications of permafrost melt, for example, we not only need to be able to understand the system as it is now, but also to project its behavior into the future. How do we do this?

Current evidence suggests that complex relational reasoning presents challenges even for adults. For example, undergraduates have considerable difficulty detecting higher-order causal patterns such as *positive feedback* and *negative feedback*, two important examples of complex relational systems (Rottman, Gentner, & Goldwater, 2012; see also Day, Motz, & Goldstone, 2015) and also the focus of the present paper. Expertise in identifying these and other causal patterns can develop, either through exposure to the same patterns across a range of domains (Rottman et al., 2012) or through a scaffolded process of comparing examples (Goldwater & Gentner, 2015). Considerable work has also examined the kinds of information that people use to make inferences about other types of causal structure in the world (Lagnado, Waldmann, Hagmayer, & Sloman, 2007; Sloman, 2005), and has detailed the challenges posed by complex systems in particular (Chi, Roscoe, Slotta, Roy, & Chase, 2012; Jacobson, Kapur, So, & Lee, 2011). An important open question, however, concerns the nature of the representations that people form as they reason about causal structure. What is the format of these representations and, moreover, how can we gain insight into them?

Possible clues come from research on how people understand complex systems more generally. Much previous research has investigated how people understand mechanical processes with multiple causal components, such as sets of gears and pulleys. A major finding of this line of work is that people often develop mental models of the system that are visuospatial in nature (Gentner & Stevens, 1983; Hegarty, 2004). One line of evidence for the visuospatial character of these models is that, when reasoning about or explaining such systems, people often produce diagrams (Forbus, Usher, Lovett, & Wetzel, 2011; Novick, 2001; Tversky, 2011) or gestures (e.g., Kang, Tversky, & Black, 2014; Nathan & Martinez, 2015; Schwartz & Black, 1996). Based on such observations, it seems plausible that people develop spatial mental models of other types of complex systems, such as the causal patterns under consideration here. However, there is a crucial difference between mechanical systems and positive and negative feedback systems. Positive feedback and negative feedback are consummate abstractions. They are relational patterns that may sometimes be instantiated in mechanical or concretely spatial systems—e.g., a flush valve toilet, which is an example of a negative feedback system—but their relational essence transcends any one concrete instantiation. It might thus seem unhelpful, or even counterproductive, to recruit visuospatial reasoning processes when thinking about such pure abstractions.

At the same time, a separate line of research has investigated how spatial concepts provide a foundation for more abstract ideas. This tendency can be seen in the spatial words and grammatical structures people draw on to talk about abstract domains, including time and others (Brugman, 1988; Clark, 1973; Heine, 1997; Jamrozik & Gentner, 2015; Traugott, 1978). In fact, evidence has now accumulated that this is not just a linguistic phenomenon—people draw on spatial representations when reasoning online about abstract concepts, whether or not language is involved (Boroditsky, 2001; Casasanto & Bottini, 2014). One vivid and naturalistic source of evidence for the use of space in abstract reasoning comes from the gestures people produce (Cienki, 1998; McNeill, 1992; Núñez, 2008). To date, perhaps the best-studied case of abstract spatial analogy in gesture—in which space is used to represent relations that are not inherently spatial—is the representation of a temporal sequence as a line. For instance, an English speaker might reference a future event in speech while locating it in gesture to their right, tacitly implying a relation between it and other unnamed events (for a review, see Cooperrider, Núñez, & Sweetser, 2014). Such temporal gestures use a simple spatial structure—a line—to capture a simple relational structure—order along a dimension—but they raise the possibility that people may create more complex spatial structures in gesture to represent more complex relational structures.

The above observations lead us to a hypothesis about how people reason about complex relational patterns like positive and negative feedback: they may do so, at least in part, by creating abstract spatial models of the relational structures involved—that is, spatial analogies. Furthermore, if this hypothesis is correct, then gesture should provide a powerful window onto this phenomenon. Gesture is well suited to the expression of spatial ideas (Alibali, 2005), and it has been shown to reveal implicit aspects of understanding that people have difficulty verbalizing (Broaders, Cook, Mitchell, & Goldin-Meadow, 2007; Goldin-Meadow, 2003). Moreover, it has been noted that the spatial information revealed in people’s abstract gestures sometimes goes beyond what is found in the language co-produced with those gestures (Casasanto & Jasmin, 2012; Cienki, 1998).

In the present studies, we explore this spatial analogy hypothesis by having people read a lesson about two types of complex relational systems—positive and negative feedback—and then explain the key differences between them. In our materials, we attempted to encourage highly abstract representations of such systems, devoid of the kinds of imagery that might prompt concrete spatial gestures. The most interesting possibility is that gesture might nonetheless reveal spatial analogies—that is, spatial models of relational structure—even though the relations involved in positive and negative feedback are not inherently spatial.

What would constitute evidence for such spatial analogies? A first kind of evidence would come from individual gestures that represent abstract relations spatially. These might include gestures representing simple relational structures, such as the use of a downward gesture to represent the notion of a *decrease*. Though interesting, such gestures do not necessarily go beyond prior observations about gestures representing simple relational structures, such as those described previously in the domain of time. More interestingly, people could represent complex relational structures within the confines of a single gesture. To our knowledge, analogical gestures of this more complex type have not been investigated in detail. A second kind of evidence may come from how gestures cohere over the course of an explanation. People may express complex relational structure—not in a single gesture—but by building up a spatial model of that relational structure over time. Gestural models have been observed in extended descriptions of concrete spatial layouts (Emmorey, Tversky, & Taylor, 2000), but gestural models of purely abstract relationships have not been described previously. Note, however, that the abstract nature of causal systems leaves open the possibility that people might not spatialize much of anything in their explanations. After all, gesture is thought to stem from vivid visuospatial or motoric imagery (e.g., Hostetter & Alibali, 2008), which was left out of our lesson by design.

If analogical gestures do occur, a secondary question concerns how those gestures relate to what people are saying when they produce them. If people’s abstract spatial gestures are most often produced along with corresponding spatial language—such as an upward gesture to represent an increase accompanied by “rise”, or a circular gesture when describing a feedback “loop”—gesture cannot be considered a uniquely revealing source of evidence for the presence of spatial analogies. Such gestures may be triggered by local lexical effects rather than by a stable, systematic spatial model of the system (see Gentner, 2001). However, to the extent that people’s gestures represent spatial ideas not found in speech, this would suggest that gesture plays a vital role in understanding the representations people draw on when reasoning about complex relational systems.

## Study 1: Basic causal systems lesson

### Methods

The study was approved by the University of Chicago Institutional Review Board, under protocol #IRB13-0248.

#### Participants

Twenty three adults from the University of Chicago community participated for course credit or cash. Four participants were excluded from the analyses: three because their gestures were largely occluded on the video; one for producing no gestures at all. In all, data from 19 participants (10 female; mean age = 20.8 years) are reported in the analyses.

#### Materials and procedure

##### Familiarization phase

After giving consent to participate and to be videotaped, participants carried out a series of activities that served both to familiarize them with causal systems and to assess their understanding of the systems. First, participants completed an adaptation of the Ambiguous Sorting Task (AST) used previously by Rottman et al. (2012) and Goldwater and Gentner (2015). In this type of sorting task, participants are given a set of vignettes printed on index cards and are asked to sort the cards into categories. Each vignette is an example of one of several types of causal systems (e.g., positive feedback) instantiated in one of several domains (e.g., economics). Participants are also given *seed cards*—vignettes just like those that need to be sorted but which serve as anchors for the categories to be used. A key feature of the task is that the seed cards leave the relevant categories open to interpretation: a participant may correctly categorize the vignettes according to the type of causal system described (e.g., positive feedback) or, more concretely, by the domain in which that system is couched (e.g., economics). In the adaptation of the AST used here, participants were presented with three seed cards: a first unlabeled card describing the phenomenon of stock market bubbles (a positive feedback system, economics domain), a second unlabeled card describing predator-prey relationships (a negative feedback system, natural world domain), and a third card that was blank except for the word ‘other.’ Participants were then presented with 11 new vignettes and were given five minutes to sort them. The vignettes described a range of phenomena, including internet routers, perspiration, interest rates, and so on, each instantiating a causal system.

After the sorting was complete, the experimenter removed the materials and prompted the participant to explain the main difference between the different categories involved in the sorting task. Most participants at least attempted to characterize differences in causal structure between the categories. This phase is the *pre-lesson explanation*. Together, the sorting task and the pre-lesson explanation served to familiarize participants with causal systems and perhaps prepared them to get more out of the lesson they would go on to study and explain (e.g., Little & Bjork, 2016).

##### Causal systems lesson

Next, participants were given a one-page written lesson (‘Causal Systems Lesson’) explaining the differences between positive and negative feedback systems (though without using those labels). The lesson was grounded in the seed cards used in the sorting task. It explained how the stock market and predator-prey vignettes exemplify different types of causal systems (see Appendix 1). The lesson also moved beyond the particular examples, characterizing in more abstract terms how each type of system involves different relationships between causal factors. Importantly, the lesson used no concrete spatial imagery. This decision was made to encourage participants to view the systems as abstractly as possible. It is well known that people gesture to represent concrete imagery (e.g., McNeill, 1992), but our interest is in the less studied question of how people gesture when viewing a phenomenon abstractly in terms of its causal structure. Additionally, the lesson used very little abstract spatial language. Participants were instructed to study the lesson for three minutes and were told that they would later be asked to explain it to another participant.

When the three minutes were up, the experimenter removed the lesson and brought in the ‘student’ (who was actually a confederate). The experimenter then prompted the participant as follows: “Please explain the lesson you just read. Go into as much detail as possible, but focus on the differences between the two types of causal systems”. The instructions made no mention of gesture. This phase is the *post-lesson explanation*.

#### Analysis

Videos of participants’ pre- and post-lesson explanations were transcribed and analyzed using ELAN video annotation software (https://tla.mpi.nl/tools/tla-tools/elan/). Participants gestured during both explanations. However, during the pre-lesson explanations, participants were highly variable in how much they talked (and thus how much they gestured), in what they talked about, and at what level of detail. The analyses reported here thus focus on the post-lesson explanations, which were more consistent across participants.

A first step in the gesture analysis was to identify all gestures in the post-lesson explanations that were “representational” (e.g., Chu, Meyer, Foulkes, & Kita, 2013). Representational gestures depict some property of a referent (commonly called “iconic” or “metaphoric” gestures), or point to a referent’s location (commonly called “deictic” gestures). In the present data, the representational gestures were abstract in nature—that is, they used location, movement, and spatial arrangement to depict ideas that themselves had no concrete location, did not actually move, and had no visible spatial properties. Once representational gestures were identified, they were then categorized into gesture types according to the type of system element they represented. We then classified the properties of these gestures, first considered individually and then considered in relation to other gestures in the explanation. Reliability was assessed by having a second coder classify the representational gestures in five randomly selected explanations (26% of the data), and it is reported with each analysis. The judgments of the primary coder were retained for all analyses.

### Results

#### Sorting performance

Participants sorted the vignettes according to the appropriate causal structure the majority of the time (*M* = 0.62, *SD* = 0.22). The variability in sorting performance was limited, however, with three quarters of participants scoring above 50% (interquartile range = 0.50 to 0.76). This limited variability made it difficult to explore possible relations between participants’ sorting performance and their gestures, and such relations are not discussed further.

#### Spatial ideas in speech

Before analyzing the spatial ideas evident in people’s gestures, we first examined the spatial ideas evident in their speech. Based on analogy theory (Gentner, 1983; Gentner & Markman, 1997), as well as on pilot studies involving similar materials, we distinguished four elements of feedback systems that people mention in their explanations: 1) the *factors* involved in the system; 2) the *changes* those factors undergo; 3) the *causal relations* between the factors; and 4) the behavior of the *whole system*. We analyzed how often people’s references to these different aspects of the systems involved overtly spatial words. A word was considered spatial if the first dictionary definition for the word uniquely described a spatial idea, such as location, orientation, size, or motion (based on American Heritage Dictionary (5th edition); see Appendix 3 for further details). On this criterion, words such as “increase,” which has a first definition that is not uniquely spatial, were not considered spatial; however, words such as “growth,” which has a first definition referring uniquely to a change in size, were. In their post-lesson explanations, participants expressed these four system elements using spatial words an average of 6.4 (*SD* = 4.9) times per explanation.

#### Spatial ideas in gesture

##### Rates and types

**Fig. 1:**
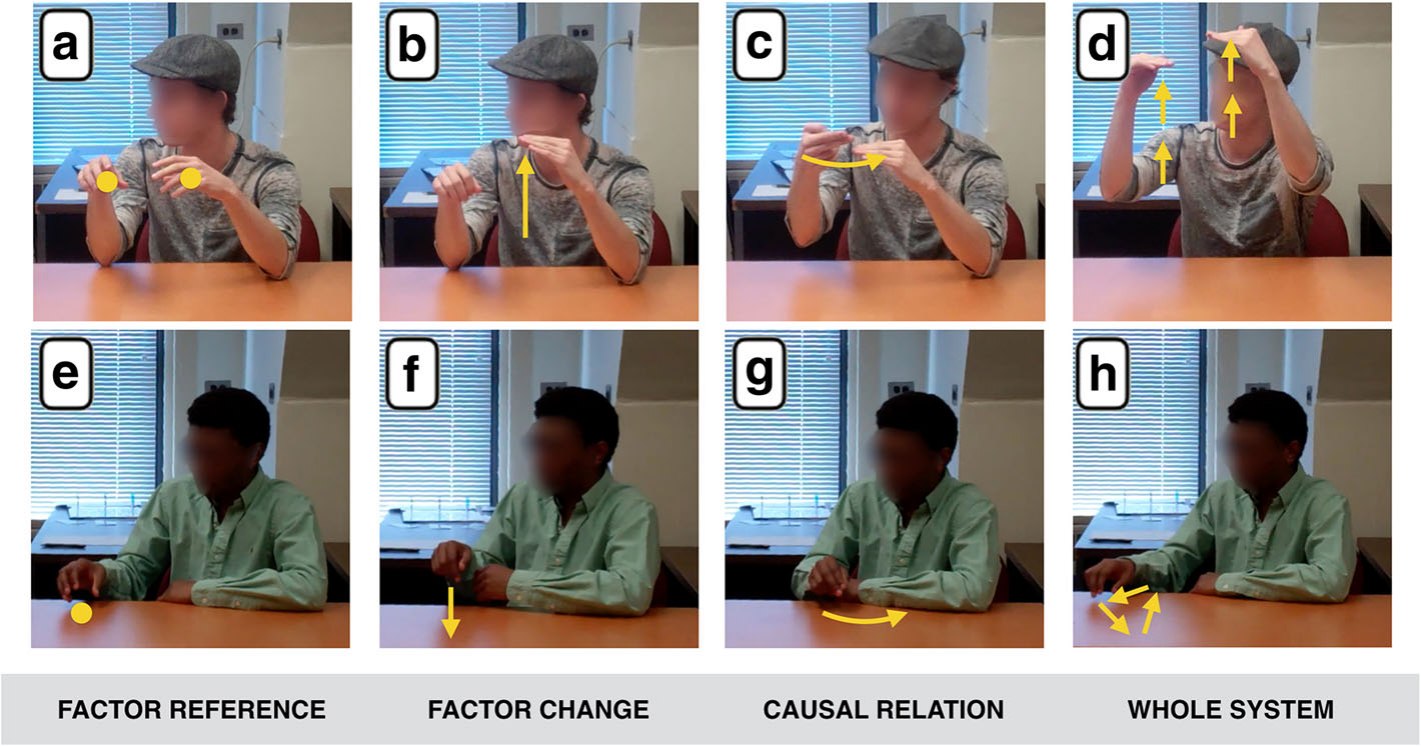
Examples of the different gesture types, taken from two participants’ explanations. *Factor reference* gestures (**a**, **e**) represent the factors as locations in space (*yellow circles*). *Factor change* gestures (**b**, **f**) represent increases and decreases as movements (*straight yellow arrows*). *Causal relation* gestures (**c**, **g**) represent causation as movement (*curved yellow arrows*). *Whole system* gestures (**d**, **h**) represent the behavior of the system as a whole and often involve multiple movement phases (*multiple yellow arrows*)

On average, participants’ post-lesson explanations lasted 88.3 seconds (*SD* = 33.1) and involved 35.0 gestures (*SD* = 13.0), for a mean rate of 24.1 (*SD* = 4.4) representational gestures per minute speaking. The abstract nature of the Causal Systems Lesson thus did not stand in the way of eliciting representational gestures. Indeed, these rates are comparable to—or higher than—gesture rates reported in studies using concrete visual stimuli (e.g., Alibali, Heath, & Myers, 2001). The participants’ gestures spatialized the four elements of feedback systems in the following ways. First, people located the factors (e.g., the predator and prey populations in the negative feedback example) by placing their gestures in space or by pointing to locations. These we call *factor reference gestures* (see Fig. 1). Second, people represented changes to the factors (e.g., an increase in the predator population) as movements. These we call *factor change gestures*. Third, people represented causal relations in the system (e.g., how the change in the predator population causes a change in the prey population) as movements, sometimes between previously established locations. These we call *causal relation gestures*. Fourth, people used movements to characterize the behavior of the system as a whole (e.g., the equilibrium that is reached in the predator-prey system). These we call *whole system gestures*. People also gestured in a variety of other ways, such as to spatially contrast the two systems they read about (with the first system in one location and the second in another), or to illustrate an aspect of the system that was not one of the intended causal factors (such as the increase in investor confidence when stock values rise).

Some gestures were unclear and could not be definitively classified into one of the four categories or the ‘other’ category. Of those gestures that both coders judged to be codable (80% of gestures analyzed), 79% (*N* = 177, Cohen’s *K* = 0.67) were assigned to the same category.

Overall, the majority (476/666, or 71%) of participants’ representational gestures fell into one of the four spatial types, with an average of 25.1 (*SD* = 9.0) such gestures per explanation.^1^ In other words, participants spatialized the system elements in gesture (25.1 per explanation) almost four times as often as they spatialized them in speech (6.4 per explanation). Further details about the counts and rates observed for each gesture type are given in Table 1. Subsequent analyses focus on those gestures that fit into the categorization scheme.

**Table 1:** Gesture counts and rates by type

	Factor reference	Factor change	Causal relation	Whole system	Other or unclear	Totals
Study 1						
Number of gestures	248	176	32	20	190	666
Mean per explanation	13.1	9.3	1.7	1.1	10.0	35.0
Proportion of participants producing type	1.00	0.95	0.68	0.47	0.79	–
Study 2						
Number of gestures	301	171	24	72	234	802
Mean per explanation	12.5	7.1	1.0	3.0	9.8	33.4
Proportion of participants producing type	1.00	0.92	0.46	0.83	0.96	–

##### Properties of gestures considered individually

We next analyzed the properties that these four gesture types exhibited, starting with the spatial axis employed in each gesture: *left-right*, *toward-away*, *up-down*, a *combination* of axes (e.g., left-right and up-down), or no single axis. Factor reference gestures were analyzed for the axis along which the factors were located; factor change gestures, causal factor gestures, and whole system gestures were analyzed for the axis along which movement was represented. Coders agreed 89% (*N* = 114, Cohen’s *K* = 0.84) of the time in judging the spatial axis involved.

The vast majority of factor reference gestures located the factors on the left-right axis (202/248, or 81%), most often with the first-mentioned factor on the left and the second-mentioned factor on the right. Factor change gestures were more spatially variable, with 21% (37/176) depicting increases and decreases as movements along the left-right axis, 29% (51/176) along the toward-away axis, 26% (45/176) along the up-down axis, and the remaining 24% (43/176) involving either some combination of these axes (e.g., simultaneously upward and rightward) or a more complex movement. Causal relation gestures most commonly (24/32, or 75%) depicted causation as movement along the left-right axis. The spatial properties of whole system gestures were much more variable (see below), with the majority not representing movement along a single axis (11/20, or 55%).

Factor reference gestures locate abstract entities in distinct spatial locations. They either do this explicitly, as when locating both factors simultaneously (Fig. 1a), or implicitly, as when locating a single factor on one side of space and implying a contrast with an unrepresented factor (Fig. 1e). The other three types of gestures represent abstract relations spatially and thus provide a first type of evidence that spatial analogies are at play. Factor change gestures represent changes in quantity (a relation between an entity at one point in time and the same entity at another point in time) as movements. Causal relation gestures represent a higher-order relation (that a change in one factor results in a change in the other factor) as an action through space. Finally, whole system gestures represent the system’s behavior through time as movement.

Importantly, these gestures differ in the complexity of the relations represented. In particular, whole system gestures summarize an entire system over time—not just a single relation such as an increase—and thus would be expected to be more spatially complex. To examine this possibility, we analyzed the type of stroke involved in the different gesture types. The stroke is the part of the gesture that is meant to represent what is being described (McNeill, 1992). The canonical gesture stroke is *simple* in that it involves a single movement phase proceeding in a single direction, but gesture strokes can also be more complex in several ways. In the case of concrete iconic gestures, complex gesture strokes might occur, for instance, when someone traces the outline of a square, shows a repetitive action such as hammering, or depicts alternating action such as two weights oscillating on a scale (for discussion of different stroke types, see McNeill, 1992, p. 308; Kita, Van Gijn, & Van der Hulst, 1998, 2014). We considered gesture strokes *complex* if they had multiple movement phases (see Fig. 1d, h). Some strokes were unclear with respect to complexity; for those gestures that both coders considered codable (97% of gestures analyzed), agreement as to whether the stroke was simple or complex was 96% (*N* = 137, *PABAK* = 0.91).^2^

**Fig. 2:**
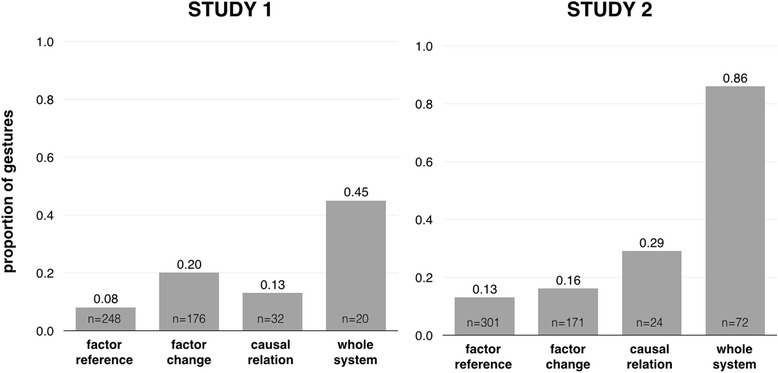
The proportion of gestures of different types that involved complex strokes

**Fig. 3:**
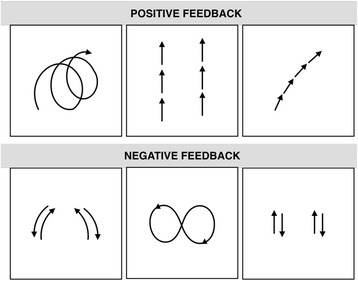
Schematic depictions of whole system gestures used to characterize the dynamics of positive and negative feedback systems

As predicted, whole system gestures involved complex strokes in 45% of cases (9/20), compared to 13% (58/456) for the other gesture types (Fig. 2). While factor change gestures, for instance, usually involve single, smooth movements along particular spatial axes, whole system gestures involve incremental upward movements, widening spirals, alternating upward and downward movements, and so on (Fig. 3). We also examined the number of participants who favored simple or complex strokes for the different gesture types. A significant number of participants favored *simple* strokes for factor reference (19/19, two-tailed exact binomial test, *p* < 0.001), factor change (17/18, two-tailed exact binomial test, *p* < 0.001), and causal relation gestures (10/13, two-tailed exact binomial test, *p* = 0.03). In contrast, a non-significant majority of participants favored *complex* strokes for their whole system gestures (5/9, two-tailed exact binomial test, *p* = 0.25).

##### Properties of gestures considered across the explanation

We next considered properties of people’s gestures across their entire explanation. Our primary interest was whether participants’ gestures were integrated into a larger analogical model—in other words, whether people produced an interconnected system of spatial gestures to represent an interconnected system of entities and relations. This kind of parallel interconnected structure is characteristic of analogical mapping (Gentner, 1983). If a participant maintains consistent, contrasting spatial locations for the factors, these gestures would be considered *model-integrated*. By contrast, if a participant locates the factors erratically from one mention to the next, these gestures would not be considered model-integrated. Further, if a participant produces a gesture representing an increase to a factor that incorporates the location of that factor as used over the course of the explanation, the gesture would be considered model-integrated. If, on the other hand, the increase was depicted in neutral space, the gesture would not be considered model-integrated. Similarly, if a participant produces a gesture representing a causal relation between two factors as a movement between the locations of the two factors as used over the course of the explanation, the gesture would be considered model-integrated (for examples, see Fig. 1c and g). Alternatively, if the gesture represented causation as a movement in neutral space, it would not be considered model-integrated. Note that we did not expect whole system gestures, which depict a high-level summary of the behavior of the system, to be closely integrated with the low-level causal details depicted in the other gestures.^3^ This gesture type was thus not included in the analysis. Importantly, model-integration goes beyond mere spatial consistency. A speaker could produce spatially consistent gestures when representing, for instance, the notion of *increase*, without ever integrating those increase gestures into a larger model.

**Fig. 4:**
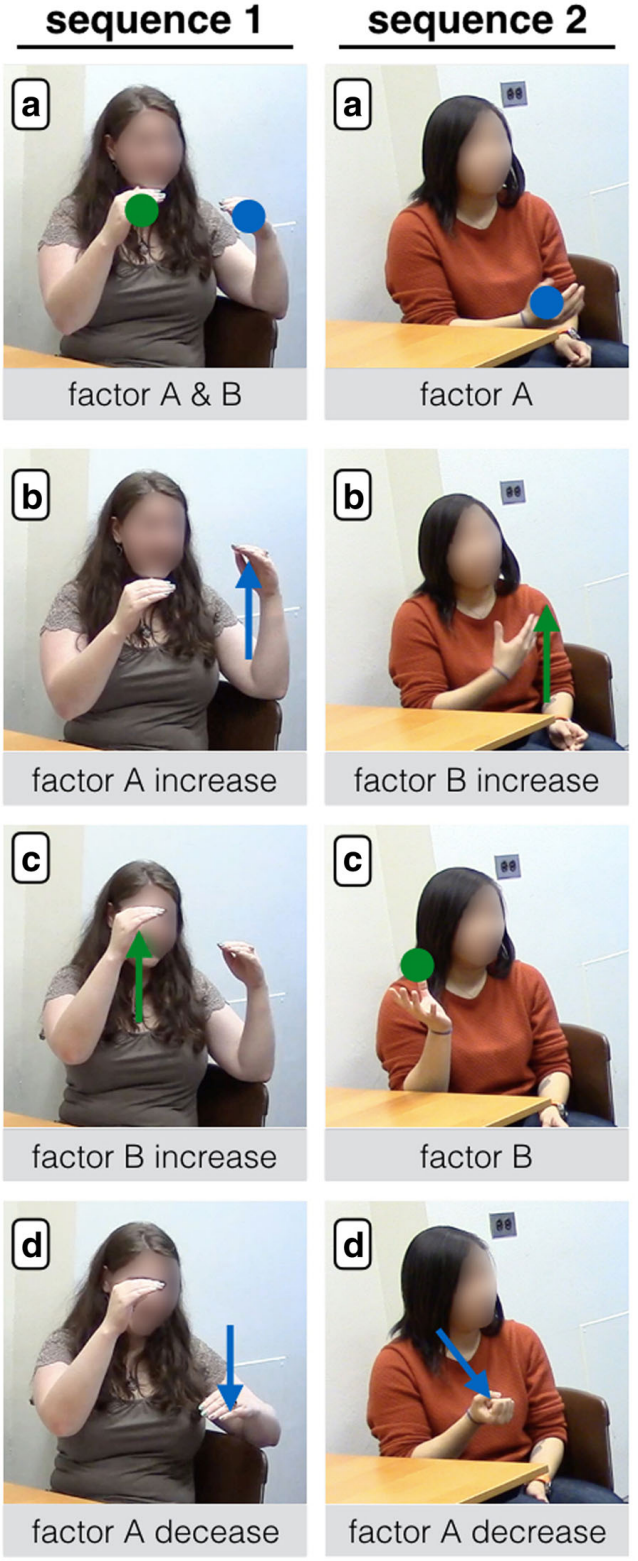
Examples of gestures that are model-integrated (sequence 1) and gestures that are not model-integrated (sequence 2) produced during explanations of negative feedback. Factor reference and factor change gestures are indicated in *blue* for factor A and in *green* for factor B. Note that the factor change gestures (the gestures with *arrows*) in sequence 1 (**b**, **c**, **d**) are consistently produced in the locations originally assigned to each factor (the *dots* in **a**). In contrast, the factor change gestures in sequence 2 (the *arrows* in **b** and **d**) are not produced in the locations assigned to the relevant factor (the *dots* in **a** and **c**)

Examples of two different gesture sequences are illustrated in Fig. 4, one with factor change gestures that are model-integrated and one with factor change gestures that are not. For some gestures it was hard to make a judgment about whether or not they were model-integrated, and they were coded as unclear. For those factor reference, factor change, and causal relation gestures that both coders judged to be codable (85% of gestures analyzed), agreement as to whether the gestures were model-integrated or not was 91% (*N* = 95, *PABAK* = 0.81).

Overall, 90% (224/248) of participants’ factor reference gestures were model-integrated (mean percentage = 90%), as were 49% (86/176) of their factor change gestures (mean percentage = 53%), and 72% (27/32) of their causal relation gestures (mean percentage = 64%). All participants (19/19) produced at least one model-integrated factor reference gesture, 84% (16/19) of participants produced at least one model-integrated factor change gesture, and 47% (9/19) produced at least one model-integrated causal relation gesture. Three participants produced *only* model-integrated gestures in their explanations: in one case, 14 gestures (three factor reference, nine factor change, two causal relation); in another, 15 gestures (five factor reference, ten factor change); and in another, 12 (all factor reference gestures). Thus, although sometimes gestures expressed unconnected flashes of spatial imagery (see Fig. 4, sequence 2), they often cohered over the course of an explanation into a systematic analogical model.

#### Gestures in relation to speech

Finally, we analyzed the relationship between participants’ gestures and the language with which they were co-produced. Most often, in 94% of cases (448/476), the gestures represented aspects of the system that were simultaneously mentioned in speech. For example, a participant would produce a factor reference gesture while referring in speech to “the first factor” or a factor change gesture while mentioning an “increase”. Interestingly, however, gestures sometimes filled in where speech left off. Of the 20 whole system gestures, three (15%) were produced without actually verbalizing the system behavior. For example, a speaker describing a positive feedback system said, “so it’s like this sort of…” trailing off in speech but providing a spatial characterization in gesture. Conveying information in gesture and not in speech occurred numerically less often for the other gesture types: in 5% of cases for factor reference gestures, 5% for factor change gestures, and 9% for causal relation gestures. The higher rate observed for whole system gestures may stem from difficulty in verbalizing the overall system dynamics. However, given the small number of observations, more data are needed to confirm this pattern.

Lastly, we investigated the relationship between the different gesture types and overt *spatial* language. If the gesture’s verbal affiliate—the word or phrase that expresses the same meaning as the gesture—included a spatial word, the gesture was classified as occurring with spatial language; otherwise, it was classified as occurring with non-spatial language. Table 2 provides examples of both spatial and non-spatial language that was co-produced with the different gesture types.

**Table 2:** Examples of spatial and non-spatial language co-produced with gestures

	Non-spatial language	Spatial language
Factor reference	“First factor”	“External variable”
	“Certain variable”	–
	“Factor A”	–
Factor change	“Increase”	“Rise”
	“Decrease”	“Go up”
	“Change”	“Go down”
Causal relation	“Influences”	“Rebounds”
	“Causes”	“Leads to”
	“Effects”	“Impacts”
Whole system	“Self-correcting”	“Negative loop”
	“Regulate each other”	“Seesaw”
	“Constant increasing”	“Building on each other”

Of the gestures that represented aspects of the system that were simultaneously mentioned in speech, the overwhelming majority (84% (403/448)) were co-produced with non-spatial language. Given the paucity of spatial ideas expressed in speech overall, as described earlier, this result is unsurprising. However, the different gesture types varied in how often they were produced with spatial language. For example, factor reference gestures, though consistently involving the left-right axis, were not once co-produced with a reference to “left” or “right” and were only co-produced with a spatial word in a single instance (“external variable”). The other gesture types did occur with spatial language, although less than half the time: factor change 32%, causal relation 45%, and whole system behavior 35%. Overall, these results confirm that much of the spatial content in people’s explanations comes out not in their words, but in their hands.

### Discussion

When explaining positive and negative feedback systems (two examples of complex relational patterns), participants recruited spatial representations in abundance. Elements of these systems were expressed spatially in gesture almost four times as often as they were expressed spatially in speech. These spatial gestures provided several types of evidence that people were drawing extensively on spatial analogy. First, participants produced individual gestures that used space to represent abstract relations. These included both simple relations, as in factor change gestures that represented increases and decreases as movements along axes, and complex relations, as in the whole system gestures used to summarize the behavior of the entire system. Second, considered in relation to each other, participants’ gestures were often integrated into larger analogical models, as when depicting a causal relationship as movement between previously established locations. Finally, the spatial analogies as revealed in gesture very often occurred without spatial language and, in some cases, without any language at all. Spatial analogies are thus pervasive in spontaneous gesture during complex relational reasoning, and are not merely a manual echo of spatial speech.

One limitation of Study 1 was that, although the lesson was largely devoid of rich imagistic content, it did include a sprinkling of abstract spatial words. For example, the words “direction” and “reversed” were used to describe changes in the system. Importantly, participants produced the same basic gesture types in their pre-lesson explanations as in their post-lesson explanations—thus, the lesson did not seem to tip participants off to the idea of spatializing aspects of the system. Nonetheless, it remains possible that the presence of spatial language increased participants’ propensity to spatialize their mental models of the system. To address this possibility, in Study 2, we removed the remaining spatial language from the Causal Systems Lesson. If the abundant use of analogical gestures in Study 1 was cued by the spatial language in our lesson, these behaviors should be considerably dampened. A second aim of Study 2 is to again examine whole system gestures (which were relatively infrequent in Study 1)—specifically, whether we will again find that they tend to involve complex strokes.

## Study 2: Causal systems lesson with spatial language removed

### Methods

The study was approved by the University of Chicago Institutional Review Board, under protocol #IRB13-0248.

#### Participants

Thirty adults from the University of Chicago community participated in exchange for course credit or cash. Six participants were excluded from the analyses for different reasons: three because of irregularities in the experimental procedure; two because many of their gestures were occluded; and one for guessing our interest in gesture during the debriefing. In all, data from 24 participants (16 female, mean age = 21.1 years) are reported in the analysis.

#### Materials and procedure

The materials and procedure were the same as in Study 1, with the following exceptions to the sorting task and the lesson.

##### Familiarization phase

The structure of the sorting task was modified in two ways from Study 1: first, participants sorted 18 vignettes instead of 11; second, another seed card was added, which was an example of a chain of causally related events. These two changes were aimed at developing a more sensitive measure of causal systems understanding than the measure used in Study 1. Participants were given eight minutes for the expanded sorting task.

##### Causal systems lesson

The written lesson was edited for Study 2 (see Appendix 2). The primary change was that spatial language was removed, at least to the extent possible. Note that some abstract uses of prepositions remain in certain stock uses (e.g., “a change in factor A,” “begins to decrease”), as it is not possible to write intelligible English without them. The *post-lesson explanation* is again the focus of our analyses (see Appendix 4 for basic information about gesture during the pre-lesson explanations).

#### Analysis

Videos were transcribed and analyzed in the same way as in Study 1, and reliability was assessed by having a second coder classify the gestures in six randomly selected explanations (25% of the data).

### Results

#### Sorting performance

Participants sorted the vignettes according to the appropriate causal structure the majority of the time (*M* = 0.75, *SD* = 0.19). As in Study 1, however, the variability in sorting performance was quite limited, with three quarters of participants scoring above 61% (interquartile range = 0.61 to 0.89). Again, because of this limited variability, we do not discuss relations between participants’ sorting performance and their gestures.

#### Spatial ideas in speech

Participants expressed the four system elements spatially in speech an average of 5.5 (*SD* = 4.2) times per explanation, a rate comparable to Study 1.

#### Spatial ideas in gesture

##### Gesture rates and types

Participant’s explanations lasted 85.5 seconds on average (*SD* = 33.1) and involved an average of 33.4 gestures (*SD* = 32.5), for a mean rate of 23.9 (*SD* = 8.9) representational gestures per minute speaking. These rates are similar to those found in Study 1. Of those gestures that both coders judged to be codable (83% of gestures analyzed), 84% (*N* = 195, Cohen’s *K* = 0.77) were assigned to the same category.

As in Study 1, the majority (568/802, or 71%) of participants’ representational gestures fell into one of the four types. Participants expressed an average of 23.7 (*SD* = 11.2) system elements spatially in their gestures. In other words, they produced more than four times as many spatial ideas in gesture (23.7 per explanation) as they did in speech (5.5 per explanation). Observed counts and rates of the different gesture types are given in Table 1. Overall, the patterns mirror those observed in Study 1, with the exception that more whole system gestures were produced, and by a greater proportion of the participants. This difference provides an opportunity to revisit provisional observations made about whole system gestures in Study 1.

##### Properties of gestures considered individually

As in Study 1, we first examined how the gestures used space, with coders agreeing about the axis used in 82% (*N* = 172, Cohen’s *K* = 0.70) of cases. The majority of factor reference gestures located the factors on the left-right axis (227/301, or 75%). Again, factor change gestures were more variable, with 20% (35/171) depicting increases and decreases as movements along the left-right axis, 18% (30/171) along the toward-away axis, 32% (55/171) along the up-down axis, and the remaining 30% (51/171) involving either some combination of these axes or a more complex movement. Causal relation gestures most commonly (16/24, or 67%) depicted causation as movement along the left-right axis. Most whole system gestures did not exhibit movement along a single axis (64/72, or 89%).

Again, we investigated whether spatially complex gestures were used to characterize relationally complex concepts. For those gestures that both coders considered codable (98% of gestures analyzed), agreement as to whether a stroke was simple or complex was 95% (*N* = 145, *PABAK* = 0.90). Whole system gestures were complex in 86% of cases (62/72), compared to 15% (74/496) for the other types combined. Thus the pattern seen in Study 1, but limited by the small number of observations, proved to be robust in Study 2. A significant number of participants favored simple strokes for factor reference gestures (23/24 participants, two-tailed exact binomial test, *p* < 0.001) and for factor change gestures (22/22 participants, two-tailed exact binomial test, *p* < 0.001). Most participants (8/11) also favored simple strokes for causal relation factors, but this bias did not reach significance (two-tailed exact binomial test, *p* = 0.16). In contrast, a highly significant number of participants (18/20) favored *complex* strokes for their whole system gestures (two-tailed exact binomial test, *p* < 0.001).

##### Properties of gestures considered across the explanation

We next analyzed gestures in relation to other gestures produced in the same explanation. Again, some gestures were considered unclear with respect to model-integration. For those factor reference, factor change, and causal relation gestures that both coders judged to be codable (66% of gestures analyzed), agreement as to whether the gestures were model-integrated or not was 82% (*N* = 101, *PABAK* = 0.64). Overall, 85% (256/301) of participant’s factor reference gestures (mean percentage = 78%), 44% (75/171) of participants’ factor change gestures (mean percentage = 42%), and 63% (15/24) of their causal relation gestures (mean percentage = 64%) were model-integrated. Moreover, 96% (23/34) of participants produced at least one model-integrated factor reference gesture, 75% (18/24) at least one model-integrated factor change gesture, and 38% (9/24) least one model-integrated causal relation gesture.

#### Gestures in relation to speech

As in Study 1, the overwhelming majority of gestures represented aspects of the system that were also mentioned in speech (546/568, or 96%), but this differed by gesture type. Gestures without speech were exceedingly rare for factor reference gestures (2%), factor change gestures (1%), and causal relation gestures (0%), but occurred numerically more frequently for whole system gestures (21%). Whole system gestures in the absence of speech were produced by eight participants. Participants turned to gesture when they had difficulty putting complex relational ideas into words.

Finally, paralleling Study 1, we found that the bulk of gestures were produced without overt spatial language (483/546, or 88%). The gesture types were associated with spatial language to differing degrees, as follows: factor reference 0%, factor change 18%, causal relation 21%, and whole system behavior 49%. Examples of spatial and non-spatial references are included in Table 2.

### Discussion

In Study 2, we modified the Causal System Lesson to remove spatial language. Despite this change—and mirroring the primary findings of Study 1—participants showed strong evidence of drawing on spatial analogy during their explanations. It appears that participants’ use of spatial analogy was spontaneous and not entirely cued by the wording of the lesson. Moreover, Study 2 yielded more cases of whole system gestures, providing stronger evidence that such gestures are more spatially complex than other gestures types.

## General discussion

In the two studies we investigated the possibility that people would spontaneously use spatial analogies when reasoning about positive and negative feedback, relational systems that are complex, widespread, and abstract in nature. As a potential window onto such hypothesized analogies, we examined the gestures people produced as they explained the main characteristics of these causal systems and the differences between them. Despite the paucity of concrete visual imagery in the lesson we provided—and, in Study 2, the lack of spatial language—when people explained the systems, they gestured at high rates. These gestures did not represent the actual locations or movements of objects—rather, they used space abstractly to represent the different factors in the system, the changes to those factors, the causal relations between the factors, and the overall dynamics of the system. Abstract system elements were thus spatialized extensively in gesture. Importantly, these elements were not often spatialized in speech, highlighting the value of observing gesture in reasoning tasks.

The gestures we observed provided several strands of evidence for the pervasiveness of spatial analogy in causal systems reasoning. A first kind of evidence comes from the gestures considered individually. Some gestures used space to represent relatively simple abstract relations. For example, factor change gestures used movements to represent changes in magnitude, and causal relation gestures used movements to depict causation as movement between the two factors. Other gestures used space to express relationally complex concepts. Whole system gestures (e.g., illustrating a balance between two changes or a pattern of incremental increase) used spatially complex movements to capture the overall behavior of the system. A second kind of evidence for the presence of spatial analogies comes from gestures considered in relation to other gestures within the same explanation. Gestures in many explanations were not merely isolated fragments of spatial imagery, but were knitted together into larger analogical models. Interestingly, the stability of such models was sometimes directly visible from the fact that people would hold an earlier gesture in place while representing another aspect of the system with the other hand (see Figs. 1 and 4). Such models exhibit a core property of analogy (Gentner, 1983) in that they involve a mapping between an interconnected system of entities and relations in one domain and a parallel system of interconnected spatial locations and movements. Of course, not everyone produced an analogical model that was fully realized (in the sense of involving all gesture types) and fully systematic (in the sense of each gesture being model-integrated). However, most participants produced multiple gesture types and at least some model-integrated gestures, and a few participants were unwaveringly systematic.

### Spatial analogies do not depend on spatial language

Research in recent decades has demonstrated a tight relationship between gesture and speech, with the two often “co-expressing” the same idea in two formats at once (McNeill, 1992). Given such observations, we might have guessed that abstract gestures would largely be used along with overt spatial words like “rise” or “loop”. Prior work has noted that abstract spatial gestures sometimes occur without any tip-off in the co-occurring speech (e.g., Casasanto & Jasmin, 2012; Cienki, 1998; Cienki & Müller, 2008), but it has been unclear from these observations whether such cases are the exception or the rule. Our results suggest that, at least in explanations of causal systems, spatial language and analogical gesture do not always go hand-in-hand. In fact, gestures were sometimes the *only* medium in which an idea surfaced at all, as when people trailed off in speech but conveyed a sophisticated spatial analogy in their hands. Moreover, the spatial analogies we observed in gesture did not depend on giving people spatial language in the lesson they were explaining. The lesson we used in Study 1 did include a small amount of abstract spatial language. However, participants produced the full suite of gesture types before ever seeing this lesson, in their pre-lesson explanations. More decisively, in Study 2, we removed the abstract spatial language from the lesson and saw no change in people’s propensity for producing spatial analogies in gesture.

### Spatial analogies in STEM

Analogies are ubiquitous in STEM fields, and spatial analogies appear to be especially so. Perhaps the best-studied examples to date are maps, scale models, and sketches (e.g., DeLoache, 2004; Forbus et al., 2011; Jee et al., 2014; Kastens & Ishikawa, 2006; Uttal & Wellman, 1989). In such cases, a set of concrete spatial relations in the world is mapped in schematic fashion to some spatial representation of that world. The analogical mapping is thus between one spatial format and another spatial format. By contrast, in the spatial analogies under examination here, the mapping is between a purely abstract set of entities and relations—including factors, changes, causation, and overall system behavior—and a set of spatial locations and spatial relations—locations, movements, movements between locations, and an overall system topology. Prior work has demonstrated that people are able to understand spatial analogies of this abstract type (Gattis & Holyoak, 1996; Gattis, 2001; Novick, 2001; see also the causal diagramming task in Goldwater & Gentner, 2015). The present work takes these prior observations one step further by showing that spatial analogies are also produced in spontaneous behavior.

How might the present findings inform classroom practice in the STEM fields? Analogical gestures are evanescent, and—like gestures generally—are a less overt medium of expression (see Goldin-Meadow, 2003), but they are nonetheless relevant for the classroom in at least two ways. First, what teachers do with their hands when introducing relational abstractions like positive and negative feedback may guide learning. Though not much is known about how analogical gestures are processed, early evidence from the domain of temporal thinking suggests that seeing abstract relational gestures has powerful consequences for downstream reasoning (Jamalian & Tversky, 2012). Second, students’ gestures may help teachers identify outright misconceptions or shaky understandings in their pupils (e.g., Alibali, Flevares, & Goldin-Meadow, 1997). As our data show, analogical gestures very often occur without spatial speech and sometimes without any speech at all. Thus, in a small group interaction, a teacher might notice that a student uses a seesawing gesture to characterize what is, in fact, a positive feedback system and then offer clarification. In sum, analogical gestures may be an important medium through which students learn from their teachers and teachers learn about their students.

An important issue for further research is whether analogical gestures might reveal individual differences in conceptual understanding, as gestures in other domains have been found to do (e.g., Church & Goldin-Meadow, 1986; Perry, Church, & Goldin-Meadow, 1988; Pine, Lufkin, & Messer, 2004). In our studies, we might have expected that students with more expert-like understanding would be more likely to produce whole system gestures or would exhibit more systematic gestural models. We did not find evidence for such relationships, but this may be due to low variability in our sorting task. In other populations, or with more intensive measures of expert-like understanding, such relationships may well emerge. Another way that gesture could reveal expert-like understanding is suggested by work on how students understand emergent processes. Chi et al. (2012) propose that to deeply understand such a system, it is not enough for a student to understand the low-level elements and dynamics of the system (in our studies, the factors, factor changes, and causal relations binding them) and the high-level dynamics of the system (in our studies, the whole system behavior); the student must also understand how the low-level behavior generates the high-level behavior. Future work could examine whether gesture provides evidence that students understand such connections between levels, using feedback systems or other types of complex systems where misconceptions are common (Chi et al., 2012; Jacobson et al., 2011).

A final point is that spatial analogies may play important roles in teaching and learning in STEM whether or not they are expressed in gesture. In the case of causal systems, such analogies may also be expressed and understood through diagrams (Ainsworth, Prain, & Tytler, 2011; Forbus et al., 2011; Goldwater & Gentner, 2015) and they can even be glimpsed—albeit in fragmentary fashion—in everyday language, as when we refer to “feedback loops” or “domino effects”. In fact, analogies may play important roles even when they go completely unexpressed: students working on problems alone, reading texts, or listening to lectures may still be engaging in backstage spatial reasoning. If space is a format in which students intuitively think about complex relational structures, then we may want to design materials, classroom activities, and assessments accordingly.

### Why are spatial analogies pervasive?

Why did our participants produce analogical gestures in such abundance? And how might the spatial analogies we see in gesture relate to the ones seen in diagrams, such as those sometimes used in STEM education? One possibility is that people create spatial analogies in gesture because of their familiarity with spatial analogies from diagrams. An alternative possibility is that people create spatial analogies in both gesture and in diagrams for the deeper reason that space is an especially intuitive format for reasoning about relational structure (see Gattis, 2001, for discussion). One reason that space is so intuitive may be that its relational structure is deeply familiar, even at a young age. Of course, these possible relationships between gestures and diagrams are not mutually exclusive: gesture and diagramming could both ultimately spring from an urge to spatialize relational structure while, at the same time, shaping each other to some degree.

The idea that space is an intuitive format for reasoning about relational structure is supported by the fact that spatial analogy crops up routinely in everyday communication. Consider an example from a 2012 public hearing on public transportation funding in San Francisco.^4^ At issue are proposed budget cuts to a program that subsidizes bus rides for low-income youth. During the hearing, a concerned commenter laments the apparent “tradeoff between maintenance and this program”. As she says “tradeoff” she holds her hands on either side of her body and rocks them up and down. In one complex stroke, the gesture represents the idea that as financial support for one (maintenance) increases, support for the other (the subsidy program) decreases. Other everyday ideas that may be illustrated in analogical gestures include relational abstractions like *reciprocity*, *tit-for-tat*, *give-and-take*, and many others (for additional discussion, see Beaudoin-Ryan & Goldin-Meadow, 2014; Cooperrider & Goldin-Meadow: When gesture becomes analogy, submitted).

The propensity to set up and use spatial analogical models has also been noted in established sign languages (Emmorey, 2014). Such a propensity is evident in the phenomenon known as “spatial modulation” (Senghas & Coppola, 2001; see also Liddell, 1995), which resembles the model-integrated gestures found in our data. In American Sign Language (ASL), for example, a verb may be said to be “spatially modulated” if it incorporates spatial information that was previously established for one or more of its arguments. Thus, to describe a person giving a book to someone else, an ASL signer would assign locations to the giver and to the recipient and then produce the sign GIVE along a path from the former to the latter. As our data show, hearing gesturers do something similar under the right circumstances (see also So, Coppola, Licciardello, & Goldin-Meadow, 2005). Together, these cases suggest that analogical gestures—and their counterpart, analogical signs—are a broader phenomenon outside the laboratory.

Space may be so intuitive as a format for relational reasoning, in fact, that people use it automatically. Analogy is sometimes thought of as the product of an effortful attempt to communicate a complex idea. However, empirical work has shown that this formulation is often misleading: analogical mapping can occur unintentionally, without any effort (Day & Gentner, 2007; see also Steen, 2008). We suggest that a similar unintentional deployment of analogy may be at work in our studies. Participants could have referred to their gestures—or to the spatial information contained therein—explicitly (e.g., “Imagine the system is like this”), but they did not. Nor did they show signs of engaging in an effortful process of design and development, as might be signaled by restarts or amendments to the spatial structure. Rather, participants appeared to construct their spatial analogies fluidly and more or less effortlessly as they articulated the relational structure they were describing.

Given the seeming automaticity with which analogical gestures were produced in our explanation task, an intriguing possibility is that they may have been helpful to the speaker. Prior work has argued that producing representational gestures aids conceptualization (e.g., Kita, 2000) and learning (e.g., Goldin-Meadow, Cook, & Mitchell, 2009), and has shown that gesturing during explanations can reduce cognitive load (e.g., Goldin-Meadow, Nusbaum, Kelly, & Wagner, 2001). To our knowledge, however, cognitive benefits of this sort have not been shown for analogical gestures of the type described here, and, if spatial analogy is indeed an effortful process, then producing analogical gestures could increase cognitive load rather than lighten it. However, one serendipitous observation lends weight to the possibility that gestures helped people reason in our studies: a handful of participants produced gestures while reading the lesson to themselves, and these gestures often formed extended sequences like those produced during the explanations. Other work has described such “co-thought gestures” in the case of concrete spatial reasoning tasks, such as mental rotation, and has shown that that they benefit reasoning (Chu & Kita, 2011; see also Jamalian, Giardino, & Tversky, 2013). Speculatively, the silent flurries of spatial gesture we observed may have helped participants encode the relational structure of the systems they were reading about.

## Conclusion

Complex relational patterns underlie diverse phenomena across the natural and social worlds. While earlier work has demonstrated the difficulties of reasoning about such patterns, much remains to be understood about the representations people bring to bear during such reasoning. Here we provide evidence that spontaneous spatial analogies pervade explanations of causal systems, and that gesture is an important—sometimes the *only*—medium through which such analogies are expressed. Indeed, evidence for such analogies would have been scarce from a verbal transcript alone. Although we have barely begun to explore this arena, it remains plausible that spatial analogies lie at the core of the human ability to understand complex relational phenomena.

## Appendix 1

### Basic Causal Systems Lesson (Study 1)

The brief paragraphs you just read give examples of different types of causal systems. Consider the first one:

When stocks are rising (a bull market), investors commonly believe that further rises are probable, and begin to buy more stocks. This buy-up increases the confidence of other investors and inspires them to buy as well. Many economists believe that this principle is occurring in the current housing boom. People are buying houses expecting the value to keep on rising.

In this example, a change in one factor (stock prices rising) causes a change in another factor (more buying activity) which then leads to a further change (stocks rising even more). More generally, in this type of causal system a change in one element in the system—factor A—causes a change in another element—factor B—which then leads to further change in factor A in the same direction as the original change. Overall, the initial change in factor A is furthered in this type of system.

This type of system contrasts with the one described in the other paragraph:

Predator/prey populations of animals sometimes follow a very predictable pattern. If the prey population increases in number, the predator population has more food and increases as well. At some point the predator population over eats and the prey population begins to decline in number. Consequently, the predator population decreases with the scarcity of available food.

In this example, a change in one factor (growth in the predator population) causes a change in another factor (decline in the prey population) which then leads to a further change (decline in the predator population). More generally, in this a change in one element of the system—factor A—causes a change in another element—factor B—which then leads to change in factor A in the opposite direction to the original change. Overall, the initial change in factor A is reversed in this type of system.

## Appendix 2

### Causal Systems Lesson with spatial language removed (Study 2)

The brief paragraphs you just read give examples of different types of causal systems. Consider the first one:

When stock values are increasing (a bull market), investors commonly believe that further increases are probable, and begin to buy more stocks. This increases the price of stocks. As stock prices increase, investors become more confident, and their rate of stock-buying increases. Many economists believe that this pattern is what causes stock market bubbles.

In this example, a change in one factor (stock prices increasing) causes a change in another factor (more buying activity), which then causes an additional change in the first factor (stock prices increasing even more). More generally, in this type of causal system a change in one element in the system—factor A—causes a change in another element—factor B—which then causes additional change in factor A that is the same as the original change. Overall, the initial change in factor A is perpetuated in this type of system.

This type of system contrasts with the one described in the following paragraph:

Predator/prey populations of animals sometimes follow a very predictable pattern. If the prey population increases in number, the predator population has more food and increases as well. At some point the predator population over eats and the prey population begins to decrease in number. Consequently, the predator population decreases with the scarcity of available food.

In this example, a change in one factor (increase in the prey population) causes a change in another factor (increase in the predator population), which then causes an additional change in the first factor (decrease in the prey population). More generally, in this type of system a change in one element of the system—factor A—causes a change in another element—factor B—which then causes a change in factor A that is the opposite of the original change. Overall, the initial change in factor A is corrected in this type of system.

## Appendix 3

### Spatial language coding procedure

1) To decide whether a gesture was accompanied by spatial language, we examined the lexical or phrasal affiliate of the gesture. For the gestures we are analyzing, the lexical or phrasal affiliate is the part that refers to the factor reference (e.g., “factor A”), the factor change (e.g., “increases”), causation (e.g., “causes”), or the whole system behavior (e.g., “oscillates”). In the case of a phrase, if any of the words is considered spatial (according to the following criteria) the gesture is considered to be “co-produced with spatial language”.

2) Co-produced language was transcribed during the gesture coding. The presence of spatial language was determined from this transcription. However, in cases of ambiguities in usage, the video was examined to consider the fuller context.

3) Words were considered spatial if the first definition listed in the American Heritage Dictionary (5th edition) was overtly and uniquely spatial in that it referred to movement, action, shape, configuration, or size.

Notes:

a) The definition consulted was for the part of speech that was actually used. Some words have spatial definitions when used as one part of speech, but non-spatial definitions (by our criteria) when used as another. For example, “reverse” is overtly spatial when used as a verb but is not when used as a noun.

b) Some words are defined in an abstract way that covers both spatial and non-spatial uses. We only consider words spatial if they do not also have a non-spatial sense given in the same definition—in other words, if they are uniquely spatial. For example, “increase” is defined in some dictionaries as “become or make greater in size, amount, intensity, or degree”. This one definition encompasses both overtly spatial (size) and non-spatial (amount, intensity, and degree) meanings.

c) In some cases, spatial words are used as part of conventional phrases that have holistic meanings. This includes, for instance, “turn” in the phrase “in turn”. These were not considered spatial unless the phrase itself has a spatial meaning. Spatial prepositions (“in,” “on,” “to”, etc.) were not considered spatial when they are part of stock phrases. For example, a “change *in*” or a “change *to*” are not considered spatial.

d) In case of ambiguity in the spatiality of the definition, example utterances were consulted.

e) If the American Heritage Dictionary (5th edition) did not list a separate definition for a common phrase, Google’s built in dictionary was consulted.

## Appendix 4

**Table 3:** Gesture counts and rates by type during pre-lesson explanations

	Factor reference	Factor change	Causal relation	Whole system	Other or unclear	Totals
Study 1 — Pre-lesson explanations						
Number of gestures	28	47	1	34	82	192
Mean per explanation	1.5	2.5	0.1	1.8	4.3	10.1
Proportion of participants producing type	0.58	0.74	0.05	0.68	1.00	–
Study 2 — Pre-lesson explanations						
Number of gestures	98	29	6	81	149	363
Mean per explanation	4.1	1.2	0.3	3.4	6.2	15.1
Proportion of participants producing type	0.75	0.50	0.21	0.79	1.00	–

## Footnotes

1 Participants also produced these same four gesture types in their pre-lesson explanations (see Appendix 4). However, we do not analyze these explanations in detail because they were shorter and more variable in terms of what participants talked and gestured about.

2 For two of our coding categories—stroke complexity and model integration—we report the Prevalence-Adjusted Bias-Adjusted Kappa, or *PABAK*. In these cases, one of the two coding categories is much more prevalent than the other, which complicates the interpretation of Cohen’s Kappa (Byrt, Bishop, & Carlin, 1993).

3 As noted by other researchers, systems often exhibit behavior at a zoomed-out, macro-level that is qualitatively distinct from the behavior they exhibit at a zoomed-in, micro-level. For discussion, see Chi et al. (2012) and Smith and Gentner (2014).

4 The example comes from an online database, the TV News Archive, and can be viewed at the following link (accessed 16 June 2016): https://archive.org/details/SFGTV_20120422_210000#start/180/end/240.
